# Pediatrics

**Published:** 1902-01

**Authors:** 


Pediatrics. The Hygienic and Medical Treatment of Children. By Thomas Morgan Rotch, M. D., Professor of the Diseases of Children, Harvard University. Third edition, rearranged and rewritten. Octavo, pp. 1042. Illustrated by numerous engravings in the text and by colored plates. Philadelphia and London: J. B. Lippincott Co. 1901.

The third edition of Rotch's pediatrics has been rearranged and rewritten, thus making it practically a new book. The changes that have been made add greatly to the value of the work. The opinion was forced on one in the older edition that the chapters on disease were an appendix to those on infant feeding; and while in this edition the author devotes much space to the subject, a great deal of useless matter has been cut out. The advances in diseases of the blood, in bacteriology and in infant feeding are incorporated in the present volume, several new colored plates and radiographs are added, and these and other changes make the work an interesting and valuable contribution to the literature of pediatrics.

The price has been redueed from $6.50 to $6 for the cloth bound book. M. J. F.

